# Serum small RNAs in metastatic colorectal cancer predict response to chemotherapy and characterize high-risk patients

**DOI:** 10.1186/s12943-024-02042-7

**Published:** 2024-06-27

**Authors:** Robin Mjelle, Are K. Kristensen, Tora S. Solheim, Ganna S. Westvik, Hege Elvebakken, Eva Hofsli

**Affiliations:** 1 Department of Cancer and Molecular Medicine, NTNU, Norwegian University of Science and Technology, Laboratoriesenteret 4. Etg Erling Skjalgssons Gate 1 7030, Trondheim, Norway; 2 Department of Pathology, St.Olav’s Hospital, Trondheim, Norway; 3 Department of Oncology, St. Olav’s Hospital, Trondheim University Hospital, Trondheim, Norway; 4 Cancer Clinic, Levanger Hospital, Nord-Trøndelag Health Trust, Trondheim, Norway; 5 Department of Oncology, Møre Og Romsdal Hospital Trust, Åsehaugen 5, 6017, Ålesund, Norway

**Keywords:** microRNA, Colorectal cancer, Serum, Small RNA

## Abstract

Metastatic colorectal cancer (mCRC) presents significant challenges in clinical management due to its heterogeneity and variable response to treatment. In this study, we conducted comprehensive small RNA (sRNA) sequencing analyses to identify sRNA biomarkers associated with survival and treatment response in mCRC patients. We measured serum sRNAs before and after chemotherapy treatment in a discovery cohort of 189 mCRC patients. Our analysis revealed 25 microRNAs (miRNA) as significantly associated with overall survival at baseline. We found that 11 of the 25 significant miRNAs were also significant in an independent validation cohort of 20 mCRC patients, including the top five miRNAs from the discovery cohort. Importantly, all but four of the 25 significant miRNAs from the discovery cohort had hazard ratios in the same direction in the validation cohort. Among the 25 significant miRNAs, we identified the miR-320 family of miRNAs as the strongest independent prognostic marker, with high baseline levels correlating with poor survival outcomes. Furthermore, post-treatment levels of the same miRNAs were even more predictive of overall survival, emphasizing the prognostic value of serum changes in miRNA levels before and after treatment. Moreover, we observed significant changes in serum miRNAs and other sRNAs when comparing samples before and after chemotherapy, with distinct expression patterns between responders and non-responders. Leveraging these differential expression patterns, we established a serum sRNA signature that accurately predicts response to chemotherapy with an area under the curve (AUC) of 0.8. In summary, our study highlights the prognostic and predictive potential of sRNA biomarkers in mCRC, offering valuable insights into patient stratification and personalized treatment approaches.

**Supplementary Information:**

The online version contains supplementary material available at 10.1186/s12943-024-02042-7.

## Introduction

Colorectal cancer (CRC) is the third most common cancer in the world, with more than 1.9 mill new cases in 2020 and almost 940 000 deaths [1]. World-wide, the survival rate for localized disease is 94% for colon cancer and 95% for rectum cancer, whereas the same rates for patients with distant metastasis (stage IV) are 13% and 18%, respectively (http://www.cancer.org/cancer/colonandrectumcancer/detailedguide/colorectal-cancer-survival-rates). Better characterization of high-risk TNM stage IV CRC is important for providing personalized treatment that could increase survival. Post-treatment surveillance of metastatic CRC (mCRC) is mainly conducted by CT-imaging. CT-imaging performs well when detecting distant metastasis, especially in the liver, however, the accuracy is limited for small lesions [2]. Complementing image-based surveillance with blood-based biomarkers could provide a more complete disease characterization and improve precision of detecting progression.

Carcinoembryonic antigen (CEA), C-reactive protein (CRP) and Albumin are widely used prognostic markers across cancer types, and are routinely measured in CRC [3–5]. Further, studies have shown that by calculating the ratio of CRP or CEA to Albumin, the prognostic value of these markers can be improved [6].

Circulating microRNAs (miRNAs) and other small RNAs (sRNAs) are stable molecules found in serum and plasma that can infer on prognosis and diagnosis in various cancers [7]. In CRC, circulating miRNAs have previously been shown to be dysregulated, both between CRC and healthy individuals and between early and late-stage CRC [8]. In mCRC, baseline serum expression of circulating miRNAs has been shown to have additive predictive value to common clinical parameters in predicting progressive disease [9].

Our group has previously shown that serum miRNAs differ between CRC patients with different TNM stage [10]. We have also detected survival associated serum miRNAs in metastatic rectal cancer patients and shown that systemic chemotherapy affects the expression levels of risk-associated miRNAs in serum [10]. In the present study, we measured serum small RNAs before and after treatment of 189 metastatic CRC patients to identify new prognostic markers. We show that chemotherapy significantly affects the expression levels of several types of small RNAs, and that serum-miRNAs improves risk-stratification in mCRC and predict future progression after chemotherapy. The data can be browsed interactively at https://github.com/MjelleLab/mCRC

## Results

### Study population

A total of 195 patients who underwent treatment for metastatic colorectal cancer between 12th of November 2014 and 12th of December 2018 were included in the study (hereafter referred to as the discovery cohort). All patients had samples collected at inclusion and 189 patients had samples collected both at inclusion and at two months (median 63 days) evaluation. In total, 384 samples underwent small RNA-sequencing. All patients underwent chemotherapy as first-line treatment, except for two patients who received immunotherapy. Irinotecan-based regimens were most common, administered to 65% of the patients, followed by oxaliplatin-based regimens (15%), 5-fluorouracil (5-FU) (12%) monotherapy, a combination of irinotecan and oxaliplatin (6%), and immunotherapy (2 patients) (Supplementary Fig. 1A). Thirteen patients were classified as MSI, and the treatment types were generally similar for MSS and MSI patients, except for two MSI patient who received immunotherapy (Supplementary Fig. 1B). The clinical characteristics for the cohorts are described in supplementary Table 1. In addition to the aforementioned discovery cohort, the miRNA-related survival associations were validated in an independent validation cohort of 20 metastatic CRC patients [10].

### Clinical variables associated with overall survival

To investigate clinical variables with prognostic impact in metastatic CRC (mCRC) we performed Cox Proportional-Hazards (coxph) overall survival analysis of the main clinical variables at diagnosis. Performance status was the most significant clinical factor associated with overall survival, followed by CRP and CEA, of which high levels were associated with reduced overall survival (Supplementary Fig. 1C). Patients with wild type BRAF had improved survival compared to patients with mutated BRAF (Supplementary Fig. 1). The CRP/CEA to albumin (Alb) ratios has previously been investigates as an alternative marker than CRP/CEA alone. We therefore tested both (CRP/Alb) and (CEA/Alb) in our cohort and found slightly more significant associations for the combined markers than CRP or CEA alone (Supplementary Fig. 1C). Low level of albumin alone was associated with overall survival, although not significant at the 0.05 statistical threshold (*P* = 0.07). The study population was divided into five treatment categories and the overall survival probability differed depending on treatment type. Specifically, focusing on the three main treatment types, patients receiving oxaliplatin-based regimens had the longest median survival (1108 days), followed by patients receiving irinotecan-based regimens (636 days) and patients receiving 5-FU monotherapy (462 days) (Supplementary Fig. 1D). The variations in survival among treatment types may not solely reflect the efficacy of the treatments themselves, but rather the biology of the underlying disease and patient situation. Patients with advanced, unresectable disease tended to receive irinotecan-based treatment, while those with less advanced or resectable disease typically received oxaliplatin-based treatment. 5-FU monotherapy was often administered to elderly or less fit patients.

### Serum miRNA-levels at diagnosis are associated with overall survival

**Fig. 1:**
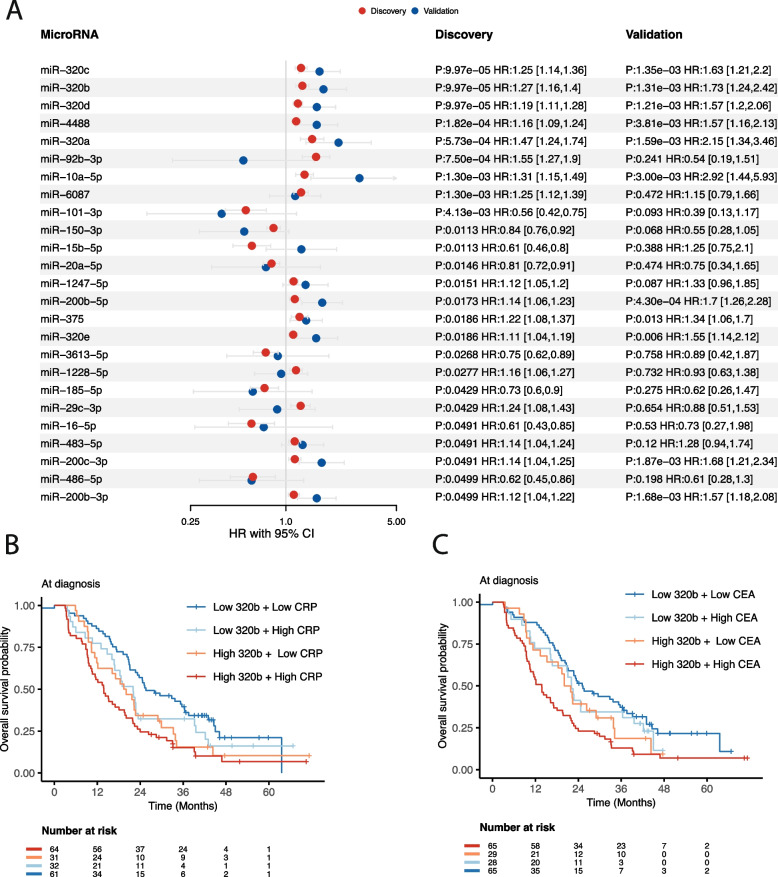
Overall survival results for miRNAs. **A** Forest plot showing the hazard ratios, confidence intervals and adjusted p-values with respect to overall survival for the significant miRNAs at diagnosis. Shown are results from the discovery and validation cohort, colored in red and blue, respectively. **B** Kaplan–Meier survival curves for miR-320b in combination with CRP. High and low miR-320b and CRP are defined as above or below the median value, respectively. The risk-table shows the number of individuals at risk for the different time intervals. **C** Similar as in A) for miR-320b and CEA. High and low CEA is defined as above or below the median CEA value

Small RNA-sequencing were performed on the whole study population, with the aim of identifying novel prognostic sRNAs related to mCRC. The sequencing statistics of the sRNA data showed that microRNAs were the most abundant RNA type comprising almost half of the reads with a fragment length peak of 22 nucleotides (Supplementary Fig. 2A-F), followed by lncRNAs, tRNAs and rRNAs. For all RNA-classes, we investigated if specific sRNAs were associated with overall survival by analyzing 195 treatment-naïve serum samples collected at diagnosis (the discovery cohort), while adjusting for sex, age, microsatellite status and treatment type. After correcting for multiple testing across the tested sRNAs, miRNAs were the only class showing significant associations, with a total of 25 significant miRNAs (Fig. 1A). 17 of the miRNAs had a positive hazard ratio and eight miRNAs had a negative hazard ratio (Fig. 1A). We sought to validate these associations by reanalyzing a sRNA sequencing dataset from 20 treatment-naïve patients with metastatic CRC, all of which had serum collected at diagnosis, like the current discovery cohort. We found that 11 of the 25 significant miRNAs were also significantly associated with overall survival in the validation cohort (coxph *p* < 0.05), and the direction of hazard ratios were generally consistent between the discovery- and validation cohort, except for four miRNAs (Fig. 1A). Importantly, the top five miRNAs from the discovery cohort were also significant in the validation cohort, including all miR-320 family of miRNAs.

### High-risk mCRC-patients have elevated levels of miR-320

Having established a panel of significant miRNAs, we aimed to refine patient stratification by integrating clinical variables with miRNA expression. C-reactive protein (CRP) and carcinoembryonic antigen (CEA) are widely used clinical indicators, typically associated with poor survival, as observed in our dataset. Hence, we examined whether combining CRP and CEA with miRNA expression enhances prognostic stratification in mCRC patients. We selected miR-320b as our focus, given its pronounced significance and higher hazard ratio both in the discovery and validation cohort compared to the other miR-320 members, which shared the same p-value. However, since all miR-320 members showed significant associations with survival, we provide detailed results for these and other miRNAs at our interactive browser https://github.com/MjelleLab/mCRC. Patients were categorized into four groups based on median miR-320b expression and CRP levels. Our findings revealed that individuals with elevated miR-320b and high CRP or CEA levels experienced the poorest survival outcomes, while those with low miR-320b and low CRP or CEA levels had the best survival (Fig. 1B-C). Notably, patients with low miR-320b and elevated CRP/CEA demonstrated significantly better survival than those with high miR-320b and elevated CRP/CEA, underscoring the independent prognostic value of miR-320b (*p* = 0.005 and *p* = 0.01 for miR-320b in multivariate models with CRP and CEA, respectively). Median survival for patients with high CRP and high miR-320b was 420 days, whereas those with high CRP and low miR-320b survived a median of 600 days, highlighting high miR-320b as a crucial subgroup with metastatic disease and significantly elevated risk (Fig. 1B). A similar trend was observed for CEA, where patients with high miR-320b and high CEA had a median survival of 418 days, compared to 638 days for those with high CEA and low miR-320b (Fig. 1C). Importantly, other members of the miR-320 family exhibited similar patterns regarding CRP/CEA levels and overall survival (results accessible through the provided interactive browser).

Although survival analyses were adjusted for treatment type, we further investigated and visualized survival outcomes for miR-320b stratified by the three primary treatment types. Our findings underscored the significant association between high miR-320b levels and overall survival across different treatment types, reinforcing miR-320b as an independent prognostic biomarker, irrespective of treatment type (Supplementary Fig. 3A-C).

### Survival associated microRNAs at two-months evaluation

After establishing the prognostic significance of miRNA expression at diagnosis in identifying high- and low-risk metastatic colorectal cancer (mCRC) patients, we aimed to assess whether miRNA expression at the two-month evaluation also holds predictive value. Conducting coxph survival analysis on miRNAs at the two-month evaluation, we identified 17 miRNAs significantly associated with overall survival (Supplementary Fig. 4A). While many of these miRNAs overlapped with those significant at diagnosis, several were uniquely significant at the evaluation point, including miR-200a-3p, miR-151b, miR-16-5p, miR-21-5p, miR-144-5p, miR-483-5p, and miR-96-5p. Although fewer miRNAs showed significant associations with overall survival at the two-month evaluation, the miR-320 family of miRNAs showed increased significance compared to the diagnosis samples. Notably, patients with low miR-320b and elevated C-reactive protein (CRP) or carcinoembryonic antigen (CEA) levels demonstrated significantly better survival than those with high miR-320b and elevated CRP levels (*p* = 2.97e-05 and *p* = 3.11e-06 for miR-320b in multivariate models with CRP and CEA, respectively), surpassing the predictive capability of the model at diagnosis (Supplementary Fig. 4B-C). Consistent with the findings in the diagnosis samples, we observed that miR-320b remained significantly associated with survival regardless of the type of treatment administered (Supplementary Fig. 3D-F).

### Major changes in serum small RNA expression two months after chemotherapy

**Fig. 2:**
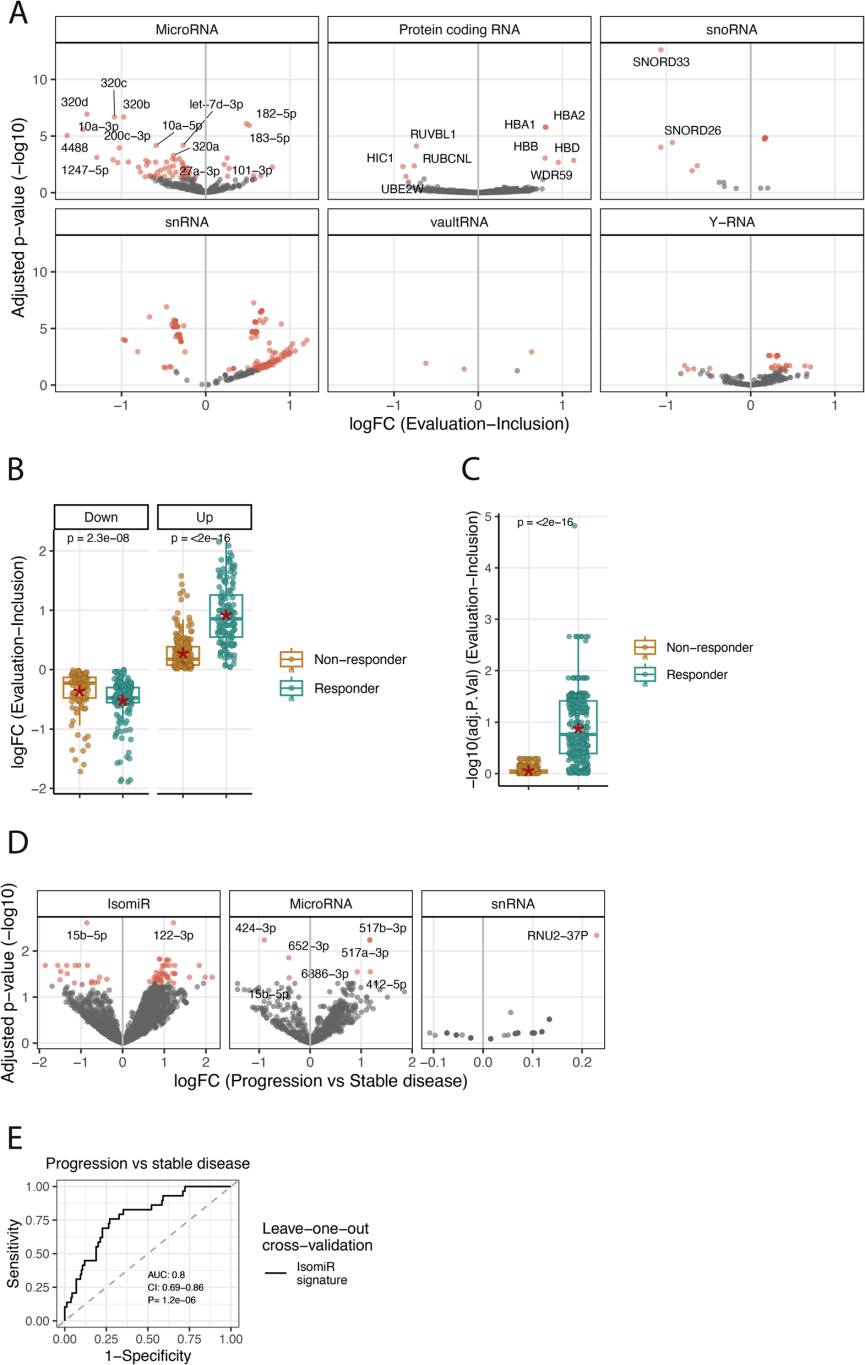
Differentially expressed miRNAs with respect to treatment. **A** Volcano plot showing differentially expressed sRNA-groups when comparing the expression before and after treatment of mCRC patients. Shown are the sRNA-groups for which significant sRNAs were detected. The x-axis shows the fold-changes values (log2) comparing the evaluation samples to the inclusion samples. The y-axis shows the inverse adjusted p-values (-log10). Selected sRNAs are indicated with name. Red indicates significance (Benjamini–Hochberg adjusted *p*-value < 0.05). **B** Differences in adjusted *p*-values for sRNAs detected as significant in A) from a differential expression analysis between two-month evaluation and diagnosis, for non-responding patients and an equal sized sampling of responding patients. The p-values are from a wilcoxon signed-rank test comparing the two groups. **C** Similar as in B) for the log-fold changes values (effect sizes). P-values calculated as in B). **D** Differentially expressed miRNAs, isomiRs and snRNAs between patients with progressive and stable disease (non-responders and responders, respectively), as defined after two months evaluation. The x-axis shows the fold-changes values (log2) between patients with progressive and stable disease. The y-axis shows the inverse adjusted p-values (-log10). Selected sRNAs are indicated with name. Red indicates significance (Benjamini–Hochberg adjusted *p*-value < 0.05) **E** ROC-curve for the leave-one-out cross-validated model predicting progressive disease at two-month evaluation based on isomiR serum-levels at diagnosis. The model is based on the top 5 differentially expressed isomiRs between responders and non-responders

To investigate alterations in serum small RNAs among metastatic colorectal cancer (mCRC) patients following chemotherapy, serum sRNAs were sequenced at diagnosis and at the two-month evaluation. Substantial changes in miRNA expression were observed when comparing samples collected before and after chemotherapy. A total of 70 miRNAs showed significant differential expression, with seven up-regulated and 63 down-regulated in the evaluation samples compared to the baseline samples (Fig. 2A). Notably, all miRNAs associated with survival demonstrated differential expression, including the miR-320 family, which ranked among the most significantly downregulated miRNAs post-treatment. Additionally, significant differential expression was observed for small RNAs across six different sRNA classes (Fig. 2A). Particularly, a pronounced up-regulation of small nuclear RNAs (snRNAs) was detected post-chemotherapy (Fig. 2A). Analysis of protein-coding RNA fragments revealed an up-regulation of hemoglobin RNAs (HBA), suggestive of pre-existing anemia in mCRC patients prior to treatment. Furthermore, inflammation-associated gene RUVBL1 exhibited higher expression levels pre-treatment compared to post-treatment (Fig. 2A). Other RNA classes displayed down-regulation of small nucleolar RNAs (snoRNAs), up-regulation of Y-RNAs, and differential expression of vaultRNAs, albeit only four vaultRNAs met our expression threshold, limiting definitive conclusions regarding the overall impact of chemotherapy on vaultRNAs. (Fig. 2A). Interestingly, one snRNA, RNU2-37P, showed reduced levels post-chemotherapy and served as a predictive marker for future progression when measured at diagnosis (Fig. 2A). Subsequently, we investigated whether serum sRNAs in responders and non-responders demonstrated differential changes from diagnosis to the first evaluation. To ensure comparable group sizes, we randomly selected 29 patients from the responder group, to match the group size of the non-responder group. Differential expression analysis between diagnosis and the two-month evaluation was performed for non-responding patients and the randomly selected responders. Comparison of p-values and effect sizes between these two groups, focusing solely on sRNAs significant in the overall analyses involving all patients, revealed significantly lower p-values and higher effect sizes in responders compared to non-responders (Fig. 2B-C), suggesting that changes in serum sRNAs post-chemotherapy may serve as potential biomarkers for treatment response.

### Small RNA levels at diagnosis identify non-responding patients

Having demonstrated the potential of miRNA levels at diagnosis and at the two-month evaluation in identifying high-risk patients, we next investigated whether miRNAs and other small RNAs at diagnosis could predict patient response to chemotherapy. We conducted a differential expression analysis comparing patients who had progressive disease (PD) two months after chemotherapy (*n* = 29) with those who showed remission or stable disease (SD) during the same period (*n* = 160). Notably, we identified eight miRNAs, 62 isomiRs (miRNA variants), and one snRNA (RNU2-37P) that showed significant differential expression between the two groups (Fig. 2D, Supplementary Table 2). These significant isomiRs were associated with 52 different miRNAs, including five belonging to miR-451a and three to let-7i-5p. Among them, miR-15b-5p emerged as a significant miRNA and isomiR, with low levels at diagnosis correlating with poor survival, consistent with its down-regulation in PD patients at diagnosis. Subsequently, we assessed whether a small RNA signature based on differentially expressed isomiRs at diagnosis could predict patients' future PD status at the two-month evaluation. We found that by combining the five most significant isomiRs from patients with PD and SD, it was possible to distinguish between them based on their isomiR levels at diagnosis. The predictive performance of this isomiR signature was validated using leave-one-out cross-validation, yielding an area-under-the-curve (AUC) of 0.80 (*P* = 1.9e-7, 95% CI: 0.71–0.88) (Fig. 2E).

## Discussion

Identifying high-risk metastatic colorectal cancer (CRC) patients is essential for tailoring personalized treatments and preventing over-treatment of non-responding individuals. In this study, we conducted the most extensive serum small RNA profiling in metastatic CRC to date. Leveraging a large patient cohort with comprehensive clinical data, we confirmed previous findings from our own and other research groups on CRC. Additionally, we identified the miR-320 family as a potential prognostic biomarker for stratifying risk groups in CRC. While miR-320 appears to be a predictive biomarker in CRC, other studies suggest that it may hold prognostic value across various cancer types. The miR-320 family has previously been linked to metastatic lung cancer, suggesting that alterations in serum levels of miR-320 could be secondary effects due to systemic immune responses in patients. [11]. Future studies that concurrently measure systemic immune responses and miRNA levels in serum could potentially clarify these associations and provide functional insights into altered serum miRNAs.

The results presented here demonstrate that miR-320 surpasses current markers such as CEA and CRP in identifying high-risk metastatic CRC patients. Inflammation is recognized as a contributing factor to cancer progression, and various inflammatory markers, including CRP, have been extensively studied in this context. CRP is synthesized by hepatocytes in response to inflammatory cytokines, notably including IL-1, TNF-α, and particularly IL-6 [12]. Baseline CRP-levels have previously been associated with CRC risk across different stage-groups [13]. A study evaluating CRC across multiple cancer types concluded that highly elevated CRP is associated with advanced disease, metastasis, and poor response prognosis [14]. In our metastatic CRC-cohorts, CRP is among the most predictive of the measured blood markers with respect to overall survival.

By accurately predicting which patients are likely to respond to chemotherapy, tailored treatment strategies to individual patients can be planned, optimizing therapeutic efficacy while reducing the burden of adverse effects on non-responders. Here we identify several sRNAs that differ between responding and non-responding patients as measured two months *before* response evaluation. These sRNAs alone or combined into a signature as demonstrated here, could potentially be used to optimize treatment to the challenging group of patients that do not respond to standard chemotherapy.

Despite extensive research on circulating miRNAs, these molecules have not yet been integrated into clinical practice. We believe that the results presented here, particularly those related to miR-320, have the potential to serve as valuable biomarkers for metastatic disease.

## Methods

### RNA isolation, library preparation and sequencing

RNA was isolated from 200uL serum using the miRNeasy Serum/Plasma Kit (ID: 217,184). Small RNA sequencing libraries were prepared using NEXTFLEX® Small RNA-Seq Kit v3 for Illumina® Platforms, following the protocol. 10.5 ul of RNA was used as input. The libraries were amplified for 18 PCR cycles. The libraries were sequenced on a HiSeq4000 machine from illumina using 50SE reads. The validation cohort was prepared and sequenced as previously described [10].

### Calibrator RNAs

Synthetic calibrator RNAs were added during the first ligation step of the library preparation protocol as previously described [10].

### Sequencing data analysis

Differentially expressed small RNAs were determined by using *limma-voom* in R. Small RNAs with at least 1 count in 50% of the data were considered in the analysis. The calcNormFactors of the calibrator RNAs were used as normalization factors in the DGE-object for the specific small RNAs.

### Survival analysis

Survival analysis for miRNAs and clinical variables were performed in R using the *coxph* function in the *survival* package. The CEA and CRP variables were converted to log2 values before conducting the *coxph* analysis. For the miRNAs, continuous expression values (cpm, log2) were used as input in *coxph*, in addition to age and sex, treatment type and microsatellite status. The results from the *coxph* were visualized using the *forestplot* package in R. The p-values for the miRNAs were adjusted for multiple testing using the benjamini–hochberg method. The Kaplan–Meier plots were calculated using the *survfit* function within the *survival* package in R. High and low miRNA expression and CRP were defined as above or below the median of the two variables. The Kaplan–Meier curved were visualized using *ggsurvplot* in R.

### Leave-one-out cross validation

The leave-one-out cross-validation (LOOCV) on the signatures was conducted in R by performing feature selection in *limma-voom* for each iteration followed by selecting the five most significant isomiRs after benjamini–hochberg adjustment, which was then used to predict the left-out sample. The predicted values were then used as input to the *roc* function in R from the *pROC* package. The specificities- and sensitivities-vectors from the *roc* function were used to plot the ROC-curves using *ggplot2*. The confidence intervals for the AUC were extracted from the *ci.auc* function and the *P*-values were calculated from the *roc.area* function. The LOOCV model is available at https://github.com/MjelleLab/Leave-one-out-cross-validation.

### Supplementary Information

Supplementary Material 1.

Supplementary Material 2.

Supplementary Material 3.

## Data Availability

Due to Norwegian law on sensitive data, raw data cannot be submitted to public repositories, however, raw data are available upon request to the corresponding author. Count matrices and metadata are available via github: https://github.com/MjelleLab/mCRC

## Abbreviations

CRC: Colorectal cancer
mCRC: Metastatic colorectal cancer
sRNA: Small RNA
miRNA: Micro RNA
CEA: Carcinoembryonic antigen
CPR: C-reactive protein
AUC: Area under the curve
MSI: Microsatellite unstable
MSS: Microsatellite stable
Coxph: Cox proportional-hazards
snRNAs: Small nuclear RNAs
snoRNAs: Small nucleolar RNAs
PD: Progressive disease
SD: Stable disease
ctDNA: Circulating tumor DNA
