# Supplementary material for: Serum small RNAs in metastatic colorectal cancer predict response to chemotherapy and characterize high-risk patients
Source: Mol Cancer. 2024 Jun 27;23:133. doi: 10.1186/s12943-024-02042-7 (PMC11209991; doi:10.1186/s12943-024-02042-7)
Supplement: Supplementary file 3 — Supplementary Material 3. [file 12943_2024_2042_MOESM3_ESM.pdf]

Supplementary information

Serum small RNAs in metastatic colorectal cancer predict response to chemotherapy and characterize high-risk patients

Robin Mjelle[1,2], Are K. Kristensen[3] , Tora S. Solheim[1,3], Ganna S. Westvik[4], Hege Elvebakken[5], Eva Hofsli[1,3]

[1]Department of Cancer and Molecular Medicine, NTNU, Norwegian University of Science and Technology, Trondheim, Norway

[2]Department of Pathology, St.Olav's Hospital, Trondheim, Norway

[3]Department of Oncology, St. Olav's Hospital, Trondheim University Hospital, Norway

[4] Cancer Clinic, Levanger Hospital, Nord-Trøndelag Health Trust, Norway

[5] Department of Oncology, Møre og Romsdal Hospital Trust

# corresponding author: Robin Mjelle (robin.mjelle@ntnu.no; robinmjelle@gmail.com). Address: Laboratoriesenteret 4. etg Erling Skjalgssons gate 1 7030 Trondheim: Phone: 0047 40234780

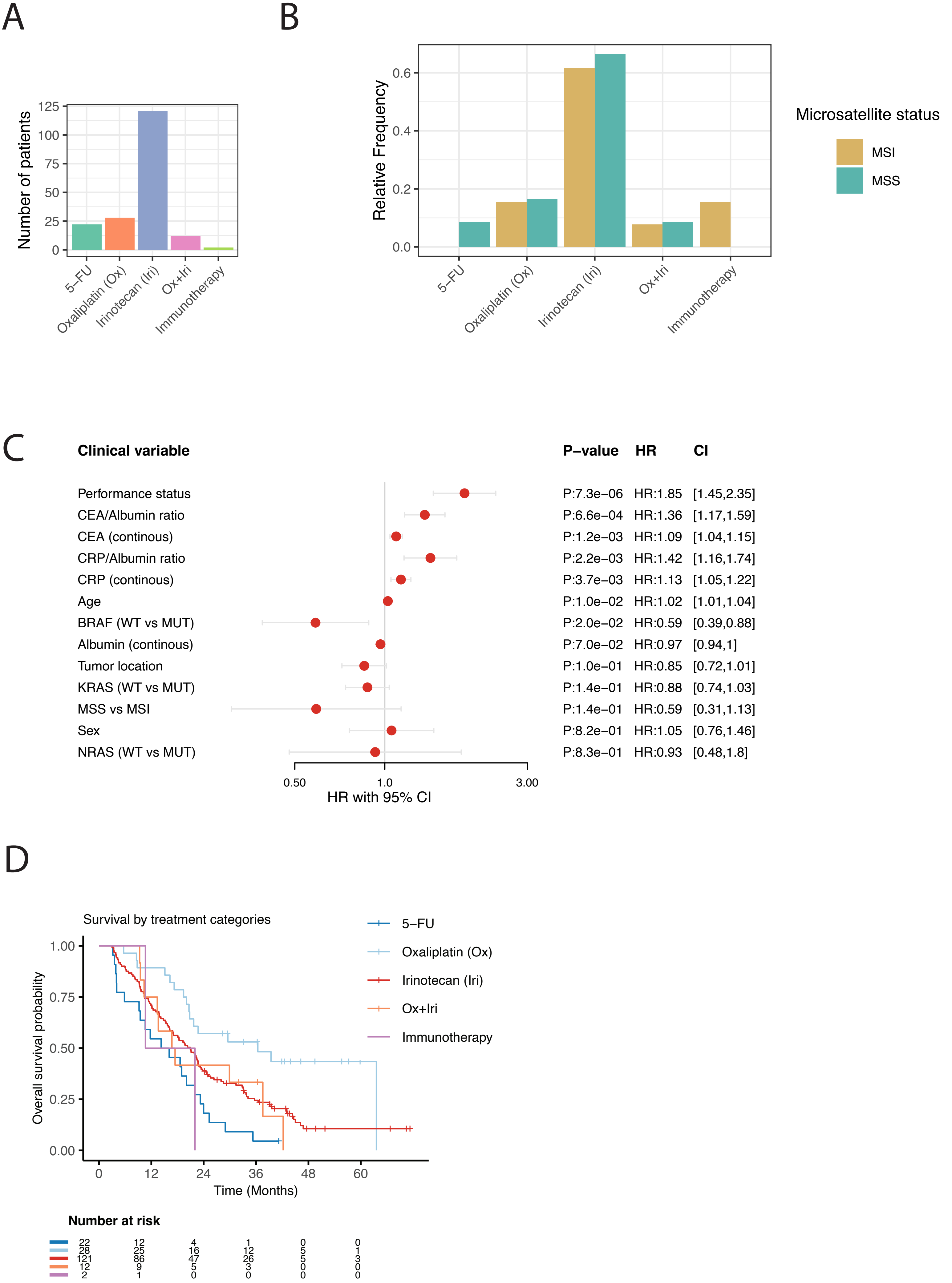
A
Number of patients
125
100
75
50
25
0
5–FU
Oxaliplatin (Ox)
Irinotecan (Iri)
Ox+Iri
Immunotherapy
B
Relative Frequency
0.6
0.4
0.2
0.0
5–FU
Oxaliplatin (Ox)
Irinotecan (Iri)
Ox+Iri
Immunotherapy
Microsatellite status
MSI
MSS
C
Clinical variable
P–value
HR
CI
Performance status P:7.3e–06 HR:1.85 [1.45,2.35]
CEA/Albumin ratio P:6.6e–04 HR:1.36 [1.17,1.59]
CEA (continous) P:1.2e–03 HR:1.09 [1.04,1.15]
CRP/Albumin ratio P:2.2e–03 HR:1.42 [1.16,1.74]
CRP (continous) P:3.7e–03 HR:1.13 [1.05,1.22]
Age P:1.0e–02 HR:1.02 [1.01,1.04]
BRAF (WT vs MUT) P:2.0e–02 HR:0.59 [0.39,0.88]
Albumin (continous) P:7.0e–02 HR:0.97 [0.94,1]
Tumor location P:1.0e–01 HR:0.85 [0.72,1.01]
KRAS (WT vs MUT) P:1.4e–01 HR:0.88 [0.74,1.03]
MSS vs MSI P:1.4e–01 HR:0.59 [0.31,1.13]
Sex P:8.2e–01 HR:1.05 [0.76,1.46]
NRAS (WT vs MUT) P:8.3e–01 HR:0.93 [0.48,1.8]
0.50
1.0
3.00
HR with 95% CI
D
Survival by treatment categories
5–FU
Oxaliplatin (Ox)
Irinotecan (Iri)
Ox+Iri
Immunotherapy
Overall survival probability
1.00
0.75
0.50
0.25
0.00
0
12
24
36
48
60
Time (Months)
Number at risk
22 12 4 1 0 0
28 25 16 12 5 1
121 86 47 26 5 3
12 9 5 3 0 0
2 1 0 0 0 0

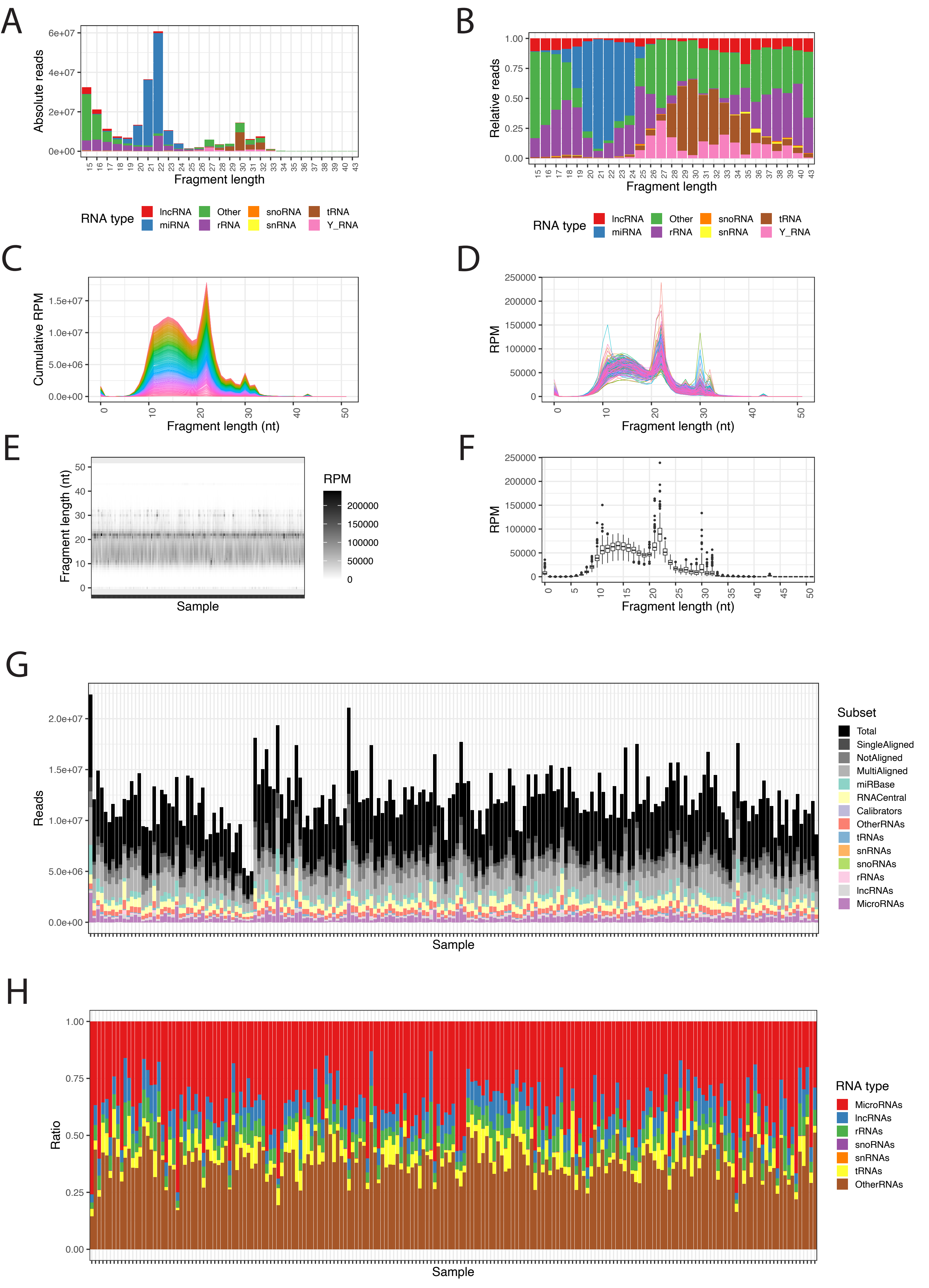

A
Absolute reads
Fragment length
RNA type
lncRNA
miRNA
Other
rRNA
snoRNA
snRNA
tRNA
Y_RNA
B
Relative reads
Fragment length
C
Cumulative RPM
Fragment length (nt)
D
RPM
Fragment length (nt)
E
Fragment length (nt)
Sample
RPM
F
RPM
Fragment length (nt)
G
Reads
Sample
Subset
Total
SingleAligned
NotAligned
MultiAligned
miRBase
RNACentral
Calibrators
OtherRNAs
tRNAs
snRNAs
snoRNAs
rRNAs
lncRNAs
MicroRNAs
H
Ratio
Sample
RNA type
MicroRNAs
lncRNAs
rRNAs
snoRNAs
snRNAs
tRNAs
OtherRNAs

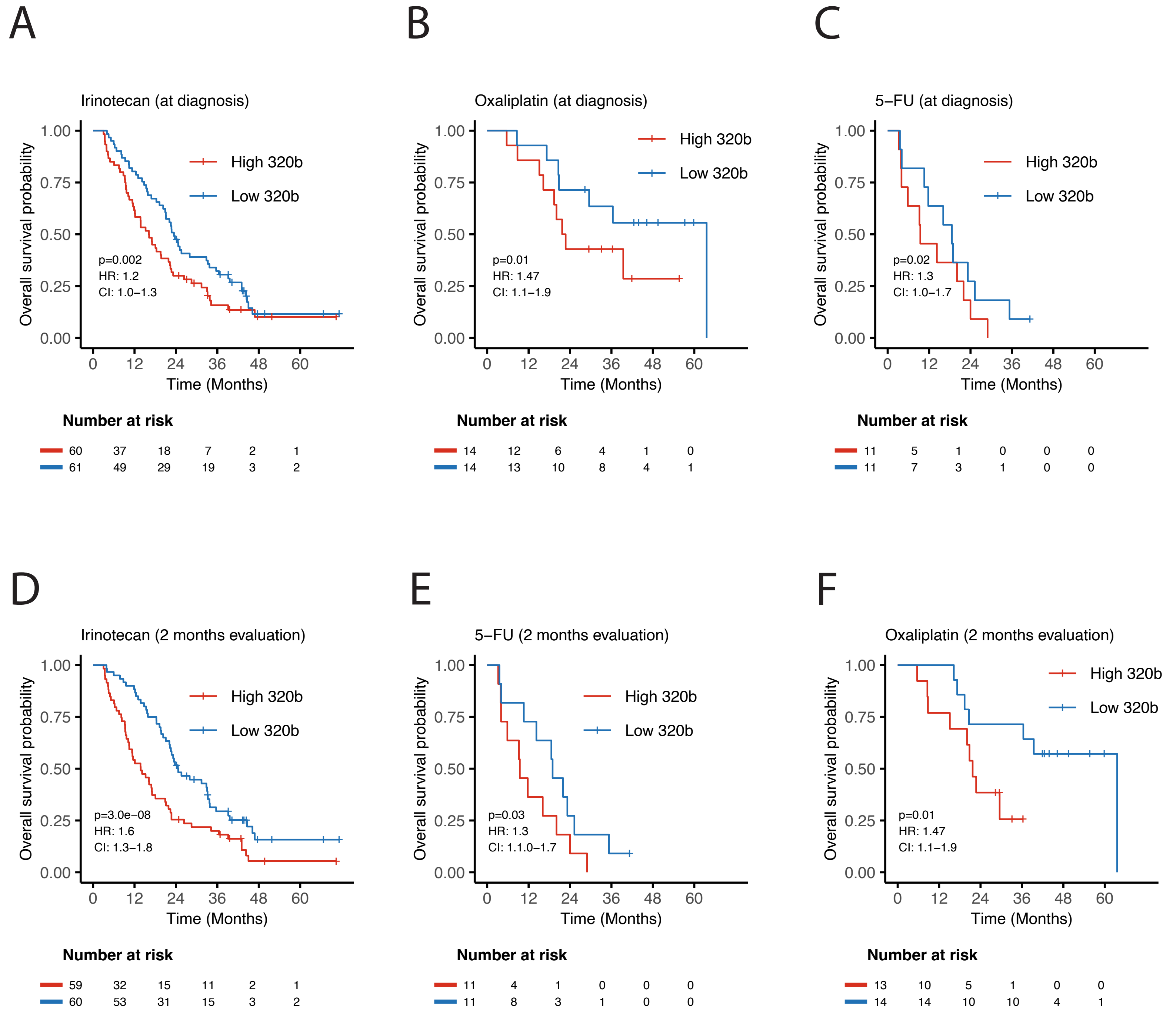
A
Irinotecan (at diagnosis)
Overall survival probability
1.00
0.75
0.50
0.25
0.00
High 320b
Low 320b
p=0.002
HR: 1.2
CI: 1.0–1.3
0 12 24 36 48 60
Time (Months)
Number at risk
60 37 18 7 2 1
61 49 29 19 3 2
B
Oxaliplatin (at diagnosis)
High 320b
Low 320b
p=0.01
HR: 1.47
CI: 1.1–1.9
Number at risk
14 12 6 4 1 0
14 13 10 8 4 1
C
5–FU (at diagnosis)
High 320b
Low 320b
p=0.02
HR: 1.3
CI: 1.0–1.7
Number at risk
11 5 1 0 0 0
11 7 3 1 0 0
D
Irinotecan (2 months evaluation)
High 320b
Low 320b
p=3.0e–08
HR: 1.6
CI: 1.3–1.8
Number at risk
59 32 15 11 2 1
60 53 31 15 3 2
E
5–FU (2 months evaluation)
High 320b
Low 320b
p=0.03
HR: 1.3
CI: 1.1.0–1.7
Number at risk
11 4 1 0 0 0
11 8 3 1 0 0
F
Oxaliplatin (2 months evaluation)
High 320b
Low 320b
p=0.01
HR: 1.47
CI: 1.1–1.9
Number at risk
13 10 5 1 0 0
14 14 10 10 4 1

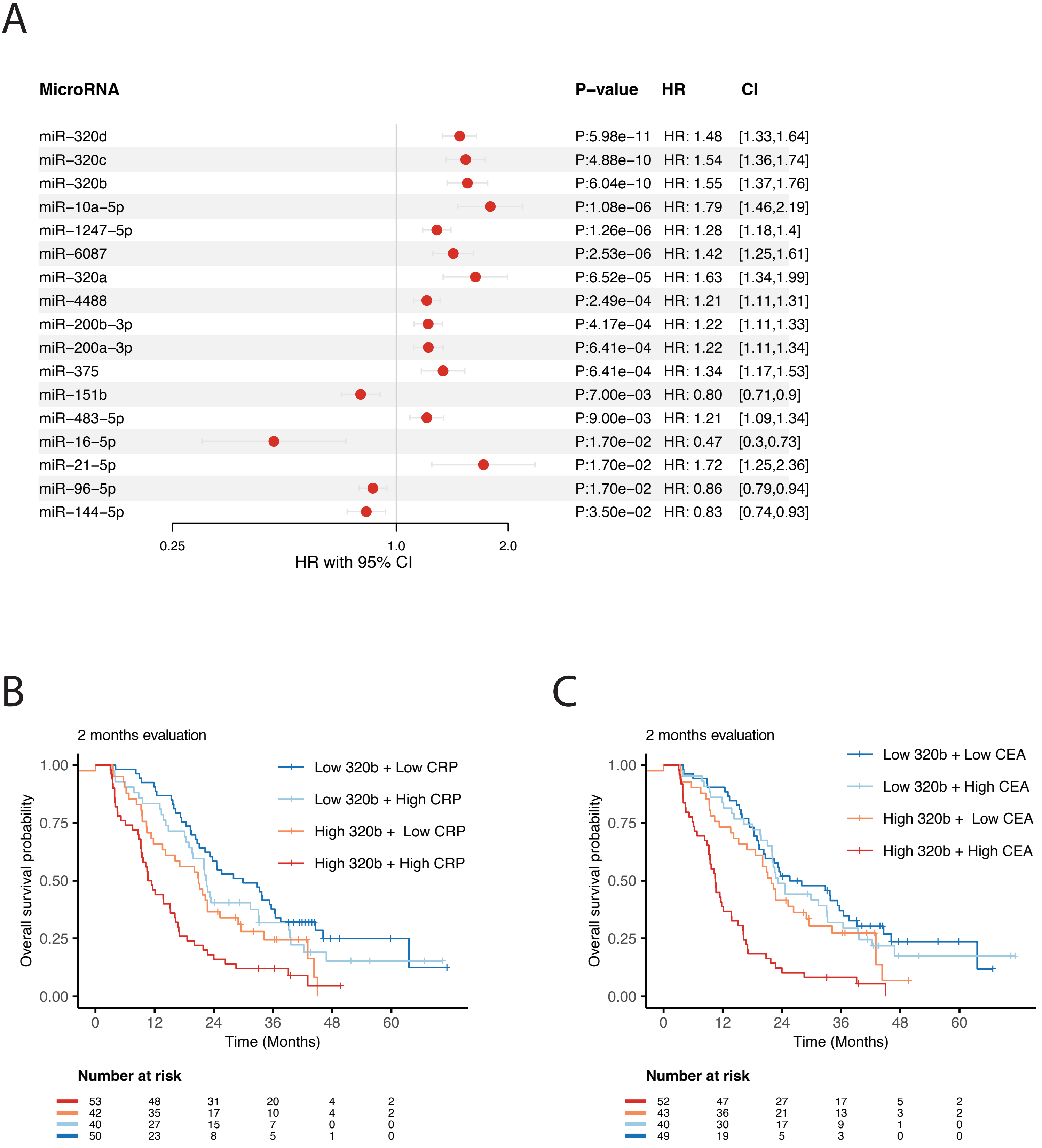
A
MicroRNA
P–value
HR
CI
miR–320d P:5.98e–11 HR: 1.48 [1.33,1.64]
miR–320c P:4.88e–10 HR: 1.54 [1.36,1.74]
miR–320b P:6.04e–10 HR: 1.55 [1.37,1.76]
miR–10a–5p P:1.08e–06 HR: 1.79 [1.46,2.19]
miR–1247–5p P:1.26e–06 HR: 1.28 [1.18,1.4]
miR–6087 P:2.53e–06 HR: 1.42 [1.25,1.61]
miR–320a P:6.52e–05 HR: 1.63 [1.34,1.99]
miR–4488 P:2.49e–04 HR: 1.21 [1.11,1.31]
miR–200b–3p P:4.17e–04 HR: 1.22 [1.11,1.33]
miR–200a–3p P:6.41e–04 HR: 1.22 [1.11,1.34]
miR–375 P:6.41e–04 HR: 1.34 [1.17,1.53]
miR–151b P:7.00e–03 HR: 0.80 [0.71,0.9]
miR–483–5p P:9.00e–03 HR: 1.21 [1.09,1.34]
miR–16–5p P:1.70e–02 HR: 0.47 [0.3,0.73]
miR–21–5p P:1.70e–02 HR: 1.72 [1.25,2.36]
miR–96–5p P:1.70e–02 HR: 0.86 [0.79,0.94]
miR–144–5p P:3.50e–02 HR: 0.83 [0.74,0.93]
0.25
1.0
2.0
HR with 95% CI
B
2 months evaluation
Low 320b + Low CRP
Low 320b + High CRP
High 320b + Low CRP
High 320b + High CRP
Overall survival probability
1.00
0.75
0.50
0.25
0.00
0 12 24 36 48 60
Time (Months)
Number at risk
53 48 31 20 4 2
42 35 17 10 4 2
40 27 15 7 0 0
50 23 8 5 1 0
C
2 months evaluation
Low 320b + Low CEA
Low 320b + High CEA
High 320b + Low CEA
High 320b + High CEA
Overall survival probability
1.00
0.75
0.50
0.25
0.00
0 12 24 36 48 60
Time (Months)
Number at risk
52 47 27 17 5 2
43 36 21 13 3 2
40 30 17 9 1 0
49 19 5 3 0 0
